# Artificial cerebrospinal fluid preserves neuronal viability and attenuates apoptosis and oxidative stress in HT22 cells

**DOI:** 10.3389/fphar.2026.1833976

**Published:** 2026-06-19

**Authors:** Lingyi Chi, Liangwen Zhang, Zhiyong Ma, Jing Gao, Xiangnan Zheng, Xuping Wang

**Affiliations:** 1 Department of Neurosurgery, Qilu Hospital, Shandong University, Jinan, Shandong, China; 2 Department of Neurosurgery, Provincial Hospital Affiliated to Shandong First Medical University, Jinan, Shandong, China; 3 National Key Laboratory of Innovation and Transformation of Collateral Disease Theory, Ministry of Education & National Health Commission Key Laboratory of Cardiovascular Remodeling and Function Research, Shandong Provincial Key Laboratory of Translational Medicine for Cardiovascular Diseases, Qilu Hospital, Shandong University, Jinan, Shandong, China; 4 Shandong Engineering Research Center of Innovative Oral Preparations, Shandong Qidu Pharmaceutical Research Institute, Zibo, Shandong, China

**Keywords:** apoptosis, artificial cerebrospinal fluid, HT22 cells, mitochondrial function, neuronal preservation, oxidative stress, p38 MAPK

## Abstract

**Introduction:**

Artificial cerebrospinal fluid (ACSF) is critical for maintaining neuronal function during surgical procedures, yet its specific neuroprotective advantages over commonly used irrigation solutions like physiological saline and phosphate-buffered saline (PBS) remain unclear.

**Methods:**

This study compared the neuronal preservation properties of ACSF, saline, and PBS using HT22 mouse hippocampal neurons, with DMEM as a control. Cell viability and apoptosis were assessed using CCK-8 assays and Annexin V-FITC/PI flow cytometry. Mitochondrial morphology and function were evaluated by transmission electron microscopy and mitochondrial membrane potential (ΔΨm) measurements. Intracellular reactive oxygen species (ROS) and calcium levels were determined using DCFH-DA and Fura-2 AM staining. Key signaling pathways were analyzed via Western blotting.

**Results:**

Results showed that ACSF significantly preserved cell viability and reduced apoptosis compared to saline and PBS. This effect was mediated by preserving mitochondrial integrity and ΔΨm, reducing the Bax/Bcl-2 ratio, suppressing ROS accumulation, and maintaining calcium homeostasis. Mechanistically, ACSF specifically inhibited the p38 MAPK/MK2 and JNK stress pathways without affecting NF-κB.

**Discussion:**

We conclude that ACSF offers superior preservation of neuronal viability compared with saline-based solutions by preserving mitochondrial function, reducing oxidative stress, and inhibiting key apoptotic pathways, supporting its preferential use in neurosurgical applications, pending *in vivo* validation.

## 1 Introduction

During neurosurgical procedures, irrigation of neural tissues is essential for maintaining a clear operative field and preserving neuronal viability. Physiological saline (0.9% sodium chloride) remains the most commonly used irrigating solution in neurosurgical practice, as described in standard neurosurgical textbooks (Handbook of Neurosurgery, 2019; Youmans and Winn Neurological Surgery, 2017) and endorsed by clinical guidelines (Carney et al., 2017; Leaper and Edmiston, 2017). Its isotonicity, ready availability, and clinical familiarity have contributed to its widespread adoption. However, emerging evidence indicates that its simplistic and non-physiological composition may place vulnerable neuronal populations at risk (Morland et al., 2016; Mahajan et al., 2024). Physiological saline lacks key components of the native neuronal milieu—including essential electrolytes (K^+^, Ca^2+^, Mg^2+^) (Lampe et al., 1995; Collins et al., 1991; Dribben et al., 2010), energy substrates such as glucose (Mergenthaler et al., 2013; Kleman et al., 2008), and intrinsic buffering systems (Chesler, 2003)—all of which are critical for maintaining ionic homeostasis, metabolic integrity, and excitability. The absence of these components can render neurons susceptible to metabolic stress, ionic imbalance, and excitotoxic injury, particularly under the mechanical and ischemic challenges inherent to neurosurgical interventions (Lampe et al., 1995; Collins et al., 1991; Dribben et al., 2010; Mergenthaler et al., 2013; Kleman et al., 2008; Chesler, 2003).

Artificial cerebrospinal fluid (ACSF) was developed to address these limitations by closely mimicking the ionic composition, osmolarity, and pH of natural CSF (Hájos and Mody, 2009; Zheng et al., 2020). Beyond reproducing physiological conditions, ACSF formulations can be further optimized to enhance preservation. In this study, we employed an ACSF with the following composition (in mEq·L^-1^ unless specified): Na^+^ 145.5, K^+^ 2.8, Mg^2+^ 2.2, Ca^2+^ 2.3, Cl^−^ 128.5, HCO_3_^−^ 23.1, phosphate 1.1; glucose 0.61 g/L (Milhorat, 1972); osmolarity ∼289 mOsm/kg; pH ∼7.3. This formulation was modified from the classic ionic profile of normal human CSF (Davson, 1967). While maintaining the concentrations of Na^+^, K^+^, Mg^2+^, HCO_3_^−^, phosphate, osmolarity, and pH, and employing the physiological glucose concentration reported for CSF, it specifically featured a reduced Ca^2+^ concentration (2.3 vs. 2.5 mEq·L^−1^) and an elevated Cl^−^ concentration (128.5 vs. 111.9 mEq·L^−1^). It was designed to stabilize neuronal membranes and minimize excitotoxicity during surgical procedures. Studies have demonstrated that in rat models of traumatic brain injury, the ACSF resulted in less cerebral edema and cellular damage compared to lactated Ringer’s and normal saline solutions, significantly attenuating increased vascular permeability (Doi et al., 2006). In a mouse brain surface irrigation model, the ACSF was more effective than normal saline in reducing hemorrhage and maintaining a clear surgical field (Fujita et al., 2010). Furthermore, in a canine model of cerebral vasospasm, the ACSF showed minimal impact on cerebral arterial diameter (Mori et al., 2013). Clinically, the specific ACSF has been applied for intraventricular irrigation and neural tissue protection, showing improved neuronal preservation and functional outcomes in surgical settings (Huang et al., 2023; Shimizu et al., 2011). Despite these documented benefits, the precise molecular mechanisms underlying ACSF-preserved neuronal viability remain poorly defined. This knowledge gap limits both the refinement of ACSF formulations and the development of evidence-based clinical protocols that maximize neuronal maintenance.

To address this unmet need, we systematically investigated the preservative effects of the ACSF in mouse hippocampal HT22 cells, directly comparing it with physiological saline and phosphate-buffered saline (PBS), a common laboratory solution used for cell washing and reagent preparation. Our study aimed to elucidate the cellular and molecular mechanisms by which ACSF maintains neuronal viability, providing a mechanistic rationale for its preferential use in neurosurgical procedures. By bridging the gap between empirical clinical benefits and molecular understanding, these findings may inform optimized irrigation strategies to enhance neuronal survival and improve patient outcomes.

## 2 Materials and methods

### 2.1 Antibodies and reagents

The following primary antibodies were used for Western blot analysis: Anti-cleaved caspase-3 (1:1000; #25128-1-AP), Anti-Bax (1:20,000; #50599-2-Ig), Anti-phospho-c-JUN (Ser63) (1:1000; #84147-1-RR), Anti-c-JUN (1:1000; #24909-1-AP), and Anti-Beta Tubulin (1:5000; #0068-1-AP) were from Proteintech. Anti-caspase-3 (1:1000; #9662), Anti-Bcl-2 (1:1000; #3498), Anti-p38 MAPK (1:1000; #8690), Anti-phospho-MK2 (Thr222) (1:1000; #3316), Anti-MK2 (1:1000; #12155), and Anti-phospho-NF-κB p65 (Ser536) (1:1000; #3033) were from Cell Signaling Technology. Anti-phospho-p38 MAPK (T180 + Y182) (1:1000; #ab4822) and Anti-NF-κB p65 (1:1000; #ab16502) were from Abcam. All chemicals were obtained from Sigma-Aldrich unless otherwise specified.

### 2.2 Solution preparation

ACSF was prepared as described previously (Milhorat, 1972). Calcium-free ACSF was prepared by omitting CaCl_2_ from the standard ACSF formulation and adding an equimolar amount of NaCl. Pyruvate-supplemented ACSF was prepared by adding sodium pyruvate to a final concentration of 1 mM. To maintain constant osmolarity, the concentration of NaCl was reduced by an equimolar amount (1 mM). Dulbecco’s modified Eagle’s medium (DMEM; high glucose, Gibco™, Cat. no. 11965-092) was obtained from Thermo Fisher Scientific (USA). PBS was purchased from Beyotime Biotechnology Co., Ltd. (Shanghai, China). Saline (0.9% NaCl) for injection was obtained from Kelun Pharmaceutical Co., Ltd. (China). Osmolarity and pH were measured using an Micro-Osmometer (Model 210, Fiske, USA) and a pH meter (Mettler Toledo, Switzerland), respectively. Ionic composition, osmotic properties, and pH of the four perfusates are presented in Supplementary Table S1.

### 2.3 Cell culture and treatments

The mouse hippocampal neuronal cell line HT22 was obtained from Cellverse Co., Ltd. (Shanghai, China) and authenticated by short tandem repeat (STR) profiling. Cells were maintained in high-glucose Dulbecco’s Modified Eagle Medium (DMEM) supplemented with 10% (v/v) fetal bovine serum (FBS) and 1% (v/v) penicillin-streptomycin at 37 °C in a humidified incubator with 5% CO_2_. Cells were subcultured using 0.25% trypsin-EDTA upon reaching 80%–90% confluence (2–3 days after plating), and all experiments were performed with cells in the logarithmic growth phase.

To assess the effects of different extracellular environments, cells were incubated for 1 h at 37 °C in either complete culture medium (DMEM) or one of the following three isotonic solutions: artificial cerebrospinal fluid (ACSF), physiological saline, or phosphate-buffered saline (PBS). Immediately prior to each experimental assay, the incubation medium was aspirated and replaced with fresh solution of the same composition to avoid confounding effects from accumulated metabolic byproducts.

### 2.4 Cell viability assessment

Cell viability was assessed using the Super-Enhanced Cell Counting Kit-8 (CCK-8; Cat. no. C0048S, Beyotime Institute of Biotechnology, China) according to the manufacturer’s instructions. Briefly, after the respective treatments, 10% (v/v) CCK-8 reagent was added directly to the cells in their existing treatment media (DMEM, ACSF, physiological saline, or PBS). Following incubation at 37 °C for 1 h, the absorbance at 450 nm was measured using a microplate reader (Synergy H1, BioTek, USA). Relative cell viability was calculated as follows [(OD_exp_ − OD_blank_) / (OD_control_ − OD_blank_)] × 100%, where the control group (untreated cells in complete DMEM) was set as 100% viability. The blank wells contained treatment medium with CCK-8 reagent but no cells.

### 2.5 Cell morphology observation

Following incubation in the respective buffers (DMEM, ACSF, physiological saline, or PBS), HT22 cells were observed using an inverted phase-contrast microscope (Olympus IX53, Japan). Morphological assessment was performed under a 20× objective to evaluate general cellular integrity, attachment, spreading, and the presence of morphological hallmarks of apoptosis. Representative images were captured from at least six random fields per condition.

### 2.6 Flow cytometry for apoptosis assay

To assess the impact of incubation media on apoptosis, HT22 cells cultured in DMEM, ACSF, saline, or PBS were subjected to analysis using an Annexin V-FITC/PI Apoptosis Detection Kit (Cat. no. C1062L, Beyotime, China) following the manufacturer’s protocol. After incubation, cells were harvested, washed, and dual-stained with Annexin V-FITC and propidium iodide (PI). Samples were analyzed immediately after staining on a BD FACSCalibur flow cytometer (BD Biosciences, USA).

Cell populations were distinguished on a dual-parameter dot plot based on Annexin V-FITC and PI signals: viable cells (Annexin V^−^/PI^−^, Q4), early apoptotic cells (Annexin V^+^/PI^−^, Q3), late apoptotic cells (Annexin V^+^/PI^+^, Q2), and necrotic cells (Annexin V^−^/PI^+^, Q1). Data were analyzed using FlowJo software (version 10.8.1, Tree Star). The total apoptotic cell percentage was defined as the sum of cells in Q2 and Q3 (late and early apoptotic populations, respectively) and was compared across experimental conditions.

### 2.7 Transmission electron microscopy (TEM) for mitochondrial ultrastructural analysis

To evaluate the effects of different incubation conditions on mitochondrial ultrastructure, HT22 cells subjected to the indicated treatments (DMEM, ACSF, physiological saline, or PBS) for 1.5 h were harvested by centrifugation. All subsequent sample processing and imaging procedures were performed at Weiya Electron Microscopy (Jinan, Shandong, China). Cell pellets were immediately fixed in 3% glutaraldehyde (pH 7.4), followed by post-fixation in 1% osmium tetroxide. Samples were then dehydrated through a graded acetone series, infiltrated with Epon 812 resin, and embedded. Ultrathin sections (∼70 nm) were prepared using an ultramicrotome (LKB-V, LKB Company, Sweden) and stained with uranyl acetate and lead citrate. The sections were examined using a transmission electron microscope (JEM-1200EX, JEOL Ltd., Japan), and images were captured with a CCD camera (MORADA-G2, Olympus Corporation, Japan).

### 2.8 Measurement of mitochondrial membrane potential

The mitochondrial membrane potential (ΔΨm) was evaluated using the JC-1 dye (Cat. No. C2006, Beyotime Institute of Biotechnology, China) and the fluorescent cationic dye Rhodamine 123 (Cat. No. R8004, Sigma-Aldrich, USA). Briefly, after the indicated treatments, cells were incubated with JC-1 working solution (1×) at 37 °C for 30 min or with Rhodamine 123 (1 μM) at 37 °C for 15 min. Following incubation, cells were washed twice with their respective treatment buffers (DMEM, ACSF, physiological saline, or PBS) to remove extracellular dye while preserving the specific extracellular conditions of each experimental group. For qualitative assessment, fluorescence images were immediately captured using an inverted fluorescence microscope (TE2000-U, Nikon, Japan) equipped with a TILLvisION digital imaging system (TILL Photonics GmbH, Germany). For quantitative analysis, fluorescence intensity was measured using a fluorescence microplate reader (Synergy H1, BioTek, USA). For JC-1, fluorescence of the monomeric (green) form was detected at excitation/emission wavelengths of 514/529 nm, and fluorescence of the aggregated (red) form was detected at 585/590 nm. The mitochondrial membrane potential was expressed as the ratio of red to green fluorescence intensity. For Rhodamine 123, fluorescence was measured at an excitation wavelength of 488 nm and an emission wavelength of 530 nm. Fluorescence values were normalized to cell number as determined by DAPI staining after image acquisition, and data were expressed as a percentage of the DMEM control group.

### 2.9 Intracellular reactive oxygen species (ROS) detection

Intracellular ROS levels were measured using the oxidant-sensitive fluorescent probe 2′,7′-dichlorodihydrofluorescein diacetate (DCFH-DA; Cat. no. S1105M, Beyotime Institute of Biotechnology, China). After treatments, cells were incubated with 10 μM DCFH-DA at 37 °C for 30 min in the dark. To remove extracellular probe, cells were then washed three times with warm PBS. For fluorescence imaging, cells were immediately observed under a fluorescence microscope (Nikon TE2000-U, Japan) to capture the distribution of the oxidized fluorescent product DCF. For quantitative analysis, fluorescence intensity was measured using a microplate reader (Synergy H1, BioTek, USA) with excitation/emission at 485/525 nm. Data from at least three independent experiments are presented as fold change relative to the control group (normalized to 1).

### 2.10 Intracellular calcium levels analysis

Intracellular calcium levels were measured using the ratiometric fluorescent indicator Fura-2 AM (Catalog number F1221, Thermo Fisher Scientific, USA). Cells were loaded with 2 μM Fura-2 AM and 0.1% Pluronic F-127 (Catalog number P6867, Thermo Fisher Scientific, USA) at 37 °C for 60 min to facilitate dye entry. After loading, cells were washed twice with their corresponding treatment buffers to remove extracellular probe. To assess calcium dynamics in response to simulated physiological changes, cells were sequentially perfused with DMEM, ACSF, physiological saline (Saline), or PBS, followed by a return to DMEM. For real-time qualitative imaging during perfusion, fluorescence was captured using a fluorescence microscope (Nikon TE2000-U, Japan) equipped with a Fura-2 filter set (excitation: 380 ± 10 nm, emission: 510 ± 20 nm). For quantitative analysis, a parallel set of samples was perfused likewise, after which fluorescence intensities were measured at excitation wavelengths of 340 nm and 380 nm (emission at 510 nm) using a microplate reader (Synergy H1, BioTek, USA). The F340/F380 ratio was calculated and is directly proportional to the intracellular free calcium concentration. For single-cell calcium imaging, Fura-2 ratiometric traces were extracted and analyzed from the same dataset. Heterogeneity of cellular responses was assessed by visual inspection of individual cell trace.

### 2.11 Western blot analysis

After treatments, HT22 cells were lysed using RIPA buffer supplemented with PMSF and protease/phosphatase inhibitors. The protein concentrations of the collected supernatants were determined with a BCA assay. Equal amounts of protein were separated by SDS-PAGE, transferred to PVDF membranes, and blocked with 5% BSA. The membranes were incubated overnight at 4 °C with primary antibodies against cleaved caspase-3, caspase-3, Bax, Bcl-2, phospho-p38 MAPK (Thr180/Tyr182), p38 MAPK, phospho-MK2 (Thr334), MK2, phospho-c-JUN (Ser63), c-JUN, phospho-NF-κB p65 (Ser536), NF-κB p65, and β-actin, followed by incubation with appropriate HRP-conjugated secondary antibodies. Protein bands were visualized via an ECL detection system. Band intensities were quantified with ImageJ software, with phosphorylated protein levels normalized to their total protein counterparts, and total protein levels normalized to β-tubulin. The Bax/Bcl-2 ratio was calculated from the normalized data.

### 2.12 Whole-cell patch-clamp measurements for electrophysiological properties

Electrophysiological recordings were performed as described in Supplementary Methods.

### 2.13 Statistical analysis

All data are presented as mean ± standard deviation (SD). Statistical analyses were performed using SPSS software (version 22.0; IBM Corporation, Armonk, NY, USA). For comparisons involving two groups, normality was first assessed using the Shapiro–Wilk test. If the data passed the normality test (p > 0.05), a two-tailed Student’s t-test was applied; otherwise, the Mann–Whitney U test was used. For multiple group comparisons, one-way analysis of variance (ANOVA) followed by Tukey’s post-hoc test was used when the assumption of homogeneity of variances was met. When this assumption was violated, the Kruskal–Wallis test followed by Dunn’s post-hoc test (with adjusted p-values) was applied. A p-value of less than 0.05 was considered statistically significant.

## 3 Results

### 3.1 ACSF maintains HT22 cell viability more effectively than physiological saline or PBS by attenuating apoptosis

**FIGURE 1:**
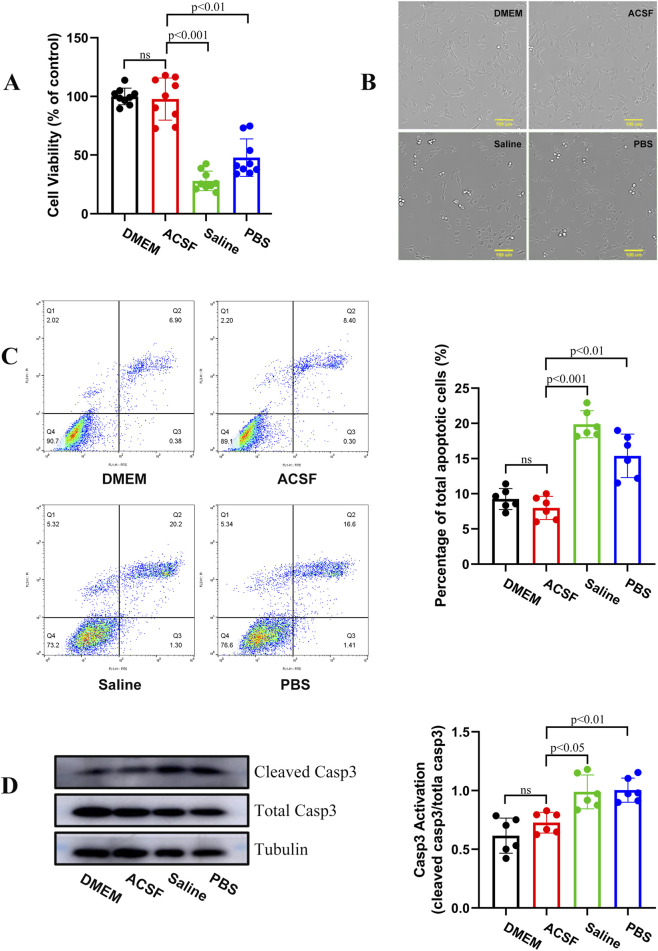
ACSF maintains HT22 cell viability more effectively than physiological saline or PBS by attenuating apoptosis. **(A)** Effects of ACSF, physiological saline and PBS on cell viability assessed by CCK-8 assay. Data are presented as mean ± SD from three independent experiments, each performed in triplicate. **(B)** Morphology of HT22 cells treated under indicated conditions. Representative images are shown. Scale bar, 100 μm (20X objective). **(C)** Flow cytometric analysis of apoptosis. Representative Annexin V/PI dot plots for each treatment group are shown. The quadrants indicate: Q4 (viable, Annexin V^−^/PI^−^), Q3 (early apoptotic, Annexin V^+^/PI^−^), Q2 (late apoptotic, Annexin V^+^/PI^+^), and Q1 (necrotic, Annexin V^−^/PI^+^). The bar graph shows the percentage of total apoptotic cells (Q2 + Q3). **(D)** Analysis of caspase-3 activation. Representative Western blot bands for cleaved caspase-3, total caspase-3, and β-tubulin (loading control) are shown. The bar graph quantifies caspase-3 activation, expressed as the ratio of cleaved to total caspase-3. Data are presented as mean ± SD from three independent experiments, each performed in duplicate. Significances were considered at p < 0.05, p < 0.01 and p < 0.001 between groups; ns, not significant (p ≥ 0.05).

CCK-8 assay showed that cell viability was significantly decreased in both the physiological saline (72.02%, p < 0.001) and PBS groups (52.21%, p < 0.01) compared to the ACSF group, while no significant difference was observed between the ACSF and DMEM control groups (Figure 1A). Morphological assessment of HT22 cells using inverted phase contrast microscopy revealed significant differences among the incubation buffers. Cells in ASCF and DMEM control displayed a predominantly normal, well-attached, and well-spread phenotype, although a subset of cells in the ASCF group showed moderate morphological alterations, including reduced spreading and slight cell body retraction. In contrast, cells incubated in saline or PBS exhibited clear features of apoptosis, such as cell rounding and shrinkage, membrane blebbing, and increased cellular debris (Figure 1B). Flow cytometry analysis consistently showed that the total apoptotic cells were significantly increased in the physiological saline and PBS groups compared to the ACSF group, by approximately 2.49-fold (p < 0.001) and 1.93-fold (p < 0.01), respectively. No significant difference was observed between the ACSF and DMEM control groups (Figure 1C). Western blot analysis further supported these findings, showing that the levels of cleaved caspase-3 were significantly elevated in the physiological saline (36.14%, p < 0.05) and PBS (38.79%, p < 0.01) groups compared to the ACSF group. Again, no statistically significant difference was detected between the ACSF and DMEM control groups (Figure 1D).

### 3.2 ACSF maintains mitochondrial integrity and prevents Bax/Bcl-2 imbalance

**FIGURE 2:**
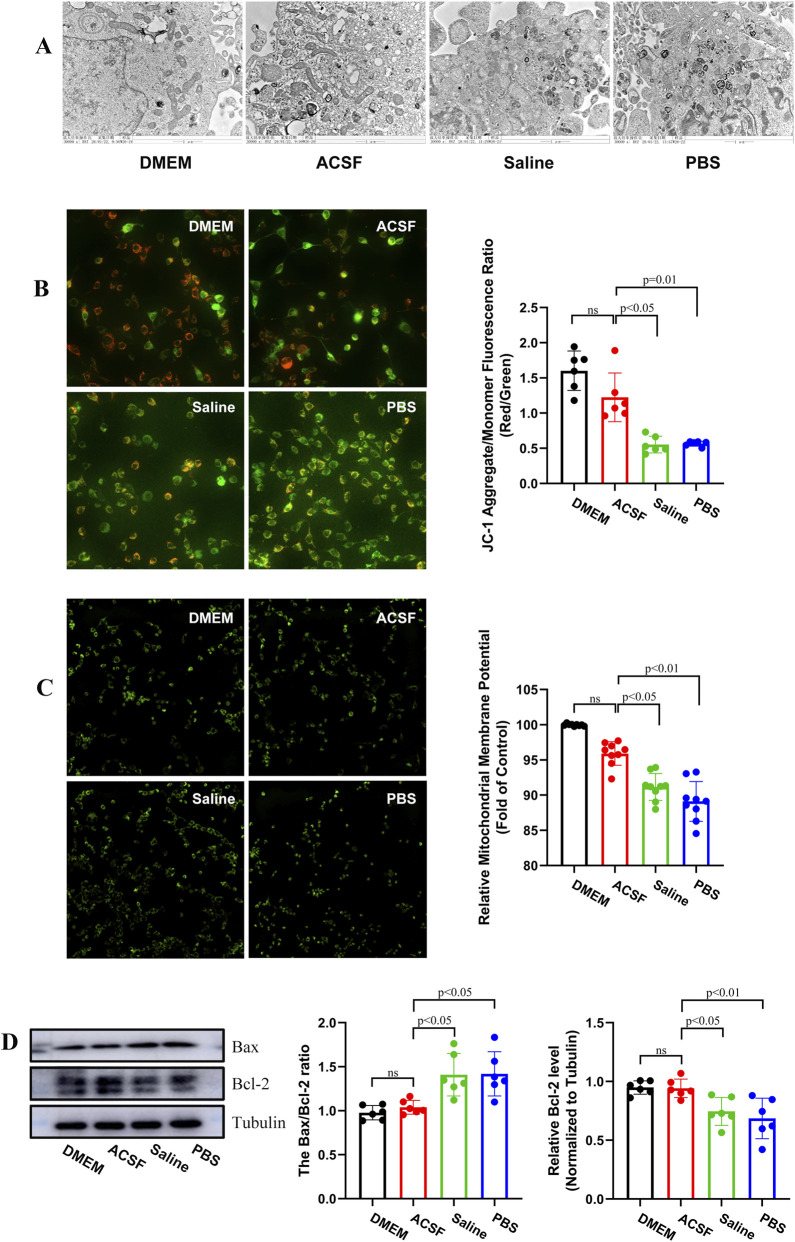
ACSF maintains HT22 cells by preventing mitochondrial damage and the associated increase in the Bax/Bcl-2 ratio. **(A)** TEM revealed distinct mitochondrial ultrastructural alterations. Representative mitochondrial images of HT22 cells incubated in DMEM, ACSF, physiological saline, or PBS for 1.5 h are shown. **(B,C)** Assessment of mitochondrial membrane potential (ΔΨm). **(B,C)** Assessment of mitochondrial membrane potential (ΔΨm). **(B)** Cells were loaded with the potentiometric dye JC-1. JC-1 staining is presented as the red/green fluorescence ratio, reflecting ΔΨm (red: J-aggregates; green: JC-1 monomers). The bar graph quantifies the relative ΔΨm as the ratio of red to green fluorescence intensity. **(C)** Cells were stained with Rhodamine 123 (Rh 123). Representative Rh123 images depict ΔΨm, with increased fluorescence indicating hyperpolarization. Data are presented as mean ± SD of triplicate samples from three independent experiments. **(D)** Evaluation of the balance between pro-apoptotic and anti-apoptotic proteins. Representative immunoblots of Bax, Bcl-2, and β-tubulin (loading control) are shown. The graph summarizes quantitative analyses of Bcl-2 expression and the Bax/Bcl-2 ratio. Data are shown as mean ± SD from three independent experiments performed in duplicate. P < 0.05 and p ≤ 0.01 were considered statistically significant.

TEM revealed distinct mitochondrial ultrastructural alterations among the different treatment groups. Mitochondria in the DMEM control group exhibited normal morphology, characterized by oval or rod-shaped structures with intact and well-organized cristae. Similarly, mitochondria in the ACSF group largely retained structural integrity, with clearly defined cristae and only occasional mild swelling, without evidence of vacuolization or cristae disruption. In contrast, cells incubated in physiological saline or PBS displayed marked mitochondrial damage, including pronounced swelling, fragmentation or complete loss of cristae, and extensive vacuolization. Frequent disruption of mitochondrial membrane integrity was observed, indicating severe mitochondrial dysfunction (Figure 2A). Consistent with these ultrastructural findings, functional assessment of mitochondrial membrane potential (ΔΨm) demonstrated a beneficial effect of ACSF. JC-1 staining revealed a slight but non-significant reduction in ΔΨm in the ACSF group compared with the DMEM control group (76.46% vs. 100%). In contrast, ΔΨm was significantly lower in the physiological saline and PBS groups compared with the ACSF group, with relative ΔΨm levels of 45.10% and 46.21%, respectively (p < 0.05 and p = 0.01) (Figure 2B). Similar results were obtained using the Rhodamine 123 assay. Although ΔΨm in the ACSF group showed a slight, non-significant reduction compared with the DMEM group (95.91% vs. 100%), a significant decrease was observed in the physiological saline and PBS groups compared with the ACSF group (91.16% and 89.11% vs. 95.91%; p < 0.05 and p < 0.01, respectively) (Figure 2C). Western blot analysis further demonstrated that Bax protein expression remained comparable across all groups, whereas Bcl-2 expression was significantly reduced in the physiological saline and PBS groups compared with the ACSF group (20.83% and 27.22% decrease, respectively; p < 0.05 and p < 0.01). As a consequence, the Bax/Bcl-2 ratio was significantly increased in these groups (1.36-fold and 1.37-fold, respectively; p < 0.05 for both comparisons). No significant differences in Bax or Bcl-2 expression, or in the Bax/Bcl-2 ratio, were observed between the ACSF and DMEM control groups (Figure 2D).

### 3.3 ACSF protects HT22 cells by inhibiting intracellular reactive oxygen species (ROS) accumulation and preventing impaired calcium signaling

**FIGURE 3:**
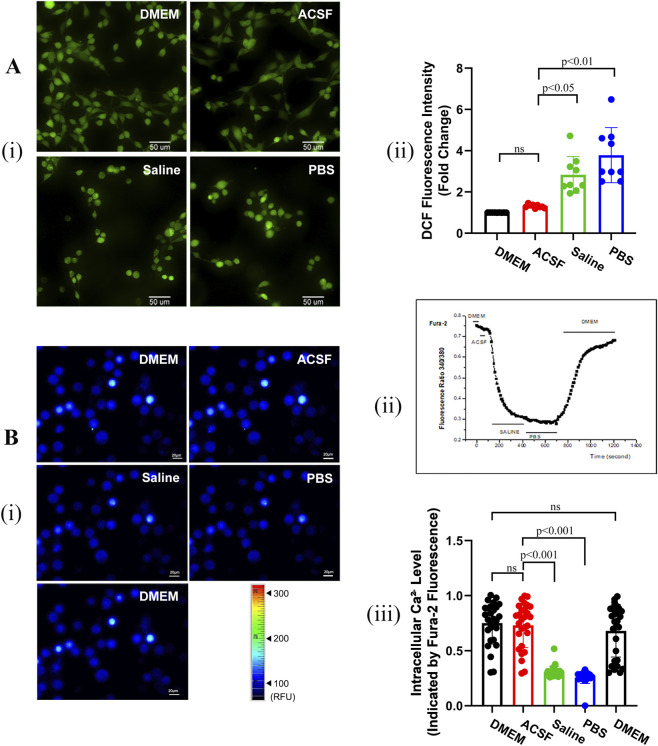
ACSF inhibits intracellular ROS accumulation and maintains calcium homeostasis in HT22 cells. **(A)** Intracellular ROS detection. (i) Representative images of HT22 cells stained with DCFH-DA. (ii) Quantification of normalized DCF fluorescence intensity. Data are presented as mean ± SD from three independent experiments, each performed in triplicate. **(B)** Intracellular calcium levels. (i) Representative pseudocolor images of cells loaded with Fura-2 AM. Fluorescence intensity is displayed using a rainbow color scale, with red indicating higher and blue indicating lower relative fluorescence units (RFU). (ii) Representative traces showing the Fura-2 fluorescence ratio (340/380 nm) over time. Cells were sequentially perfused with serum-free DMEM, ACSF, physiological saline or PBS, and finally DMEM. (iii) Summary of the Fura-2 fluorescence ratio (340/380 nm). Data are presented as mean ± SD from a total of 28 cells across four independent experiments. Statistical significance is denoted as p < 0.05, p < 0.01, and p < 0.001; ns indicates no significant difference (p ≥ 0.05).

DCFH-DA staining revealed that ROS levels, as indicated by DCF fluorescence intensity, were not significantly different between the ACSF and DMEM control groups (1.3-fold increase, p = 0.07). In contrast, a marked increase in ROS was observed in the physiological saline (2.16-fold, p < 0.05) and PBS (2.89-fold, p < 0.01) groups compared to the ACSF group (Figure 3A). In contrast to the elevated ROS levels, intracellular calcium levels (measured by Fura-2 AM) showed a concomitant significant reduction. Compared to the ACSF group, the Fura-2 fluorescence ratio decreased by 57.62% in the physiological saline (Saline) group and by 64.78% in the PBS group (both p < 0.001). No significant difference was observed between the ACSF group and the DMEM control groups (at the start and end of perfusion) (Figure 3B).

Single-cell analysis revealed substantial heterogeneity in calcium dynamics during saline or PBS exposure and subsequent recovery. In most cells, intracellular calcium levels decreased markedly upon exposure to saline or PBS and showed clear recovery upon re-introduction of Ca^2+^-containing DMEM. In contrast, a minority of cells exhibited only modest changes under the same conditions (Supplementary Figure S1). Electrophysiological analysis further showed that action potential (AP) amplitude and duration (APD) were larger in ACSF compared to PBS or saline, whereas no significant differences were found between ACSF and DMEM. Resting membrane potential (RMP) did not differ between ACSF and the other solutions (Supplementary Figure S2).

### 3.4 ACSF suppresses the excessive activation of key stress-related pathways in HT22 cells

**FIGURE 4:**
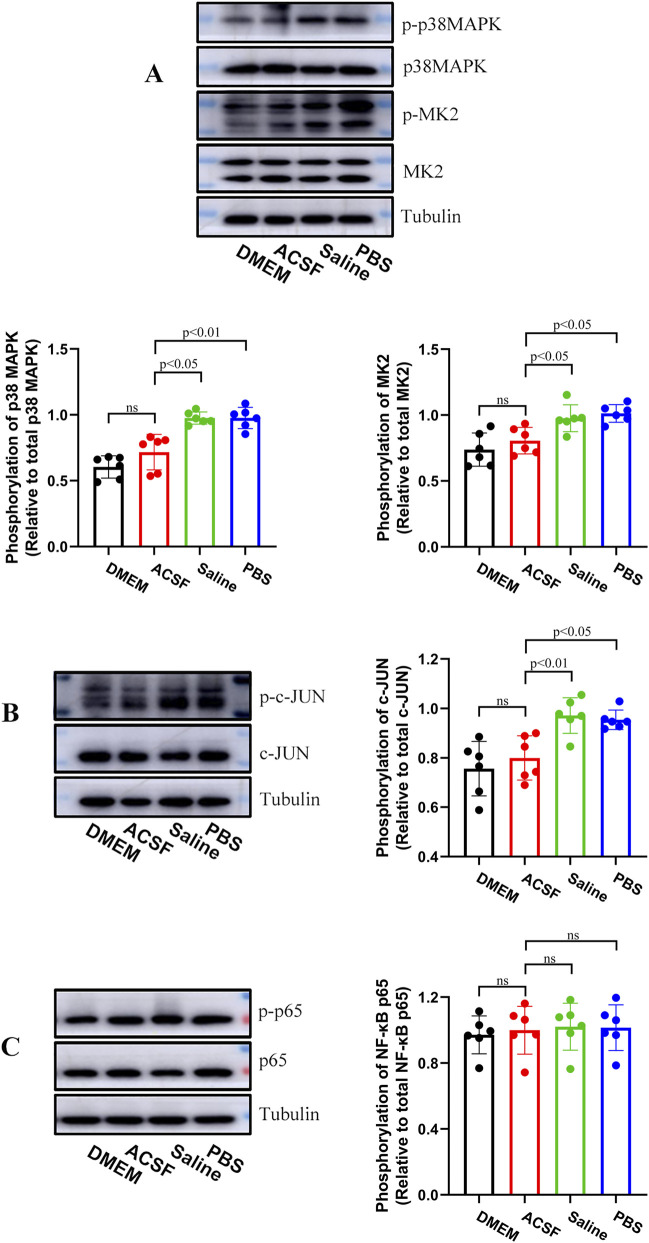
ACSF inhibits excessive activation of stress-related signaling pathways in HT22 cells. Western blot analysis was performed to evaluate the effects of ACSF on key stress-related pathways. **(A)** Pp38 MAPK pathway: Representative immunoblots and quantitative analysis of phospho-p38 MAPK (p-p38) and phospho-MK2 (p-MK2), normalized to their corresponding total protein levels. **(B)** c-JUN pathway: Representative immunoblots and quantitative analysis of phospho-c-JUN (p-c-JUN), normalized to total c-JUN. **(C)** NF-κB pathway: Representative immunoblots and quantitative analysis of phospho-NF-κB p65 (p-p65), normalized to total p65. β-Tubulin was used as a loading control. Data are presented as mean ± SD from three independent experiments, each performed in duplicate. Statistical significance is indicated as *p* < 0.05 and p < 0.01; ns denotes no significant difference (*p* ≥ 0.05).

Western blot analysis showed that the levels of phosphorylated p38 MAPK (p-p38 MAPK) and phosphorylated MK2 (p-MK2) were significantly elevated in the physiological saline (1.40-fold and 1.23-fold, respectively; p < 0.05 for both) and PBS (1.40-fold, p < 0.01 and 1.27-fold, p < 0.05, respectively) groups compared to the ACSF group. No significant differences in these proteins were observed between the ACSF and DMEM control groups (Figure 4A). Similarly, the phosphorylation level of c-JUN was significantly increased in both the physiological saline (1.22-fold, p < 0.01) and PBS (1.20-fold, p < 0.01) groups relative to the ACSF group, whereas the ACSF group again showed no significant difference from the DMEM control (Figure 4B). In contrast, the phosphorylation of NF-κB p65 was not significantly altered in either the physiological saline or PBS groups compared to the ACSF group. Likewise, no significant difference was found between the ACSF and DMEM control groups (Figure 4C).

### 3.5 Calcium in ACSF, but not pyruvate supplementation, is required for maintaining cell viability

HT22 cells were treated with standard ACSF or calcium-free ACSF under identical experimental conditions, and cell viability was assessed. The CCK-8 assay showed a significant reduction in cell viability in the calcium-free ACSF group (reduce to 86.47% of the ACSF group, p < 0.001) compared with the ACSF group (Supplementary Figure S3A). Morphological assessment using inverted phase-contrast microscopy revealed distinct changes between incubation conditions. Cells maintained in standard ACSF exhibited a predominantly normal morphology, characterized by firm attachment and a well-spread appearance. In contrast, cells exposed to calcium-free ACSF showed marked morphological alterations consistent with calcium deprivation, including reduced cell spreading, loss of cell–substrate adhesion, cell rounding, and partial detachment. An increase in floating cells and cellular debris was also observed (Supplementary Figure S3B).

In contrast, supplementation of ACSF with additional pyruvate did not significantly affect cell viability or morphology under the same experimental conditions. CCK-8 assay revealed no significant difference between the ACSF and ACSF-plus-pyruvate groups (p > 0.05), and phase-contrast microscopy showed comparable cell attachment, spreading, and overall morphology (Supplementary Figures S3C,D).

## 4 Discussion

In this study, we show that ACSF provides a better-preserved extracellular environment for HT22 hippocampal neurons than either physiological saline or PBS. Collectively, our findings support a mechanistic framework in which ACSF preserves mitochondrial integrity, thereby limiting calcium dysregulation and oxidative stress, attenuating stress-activated signaling pathways, and ultimately reducing apoptosis-associated neuronal loss.

A principal finding of this work is that ACSF significantly reduces neuronal apoptosis. This conclusion is supported by both morphological observation and flow cytometry assays, which show markedly fewer apoptotic cells in the ACSF group. Consistently, cells exposed to saline or PBS exhibited strong activation of cleaved caspase-3, the central executioner protease of apoptosis (Srinivasula et al., 1996), a response consistent with prior studies showing that ionic imbalance or non-physiological extracellular solutions rapidly trigger neuronal apoptosis (Bortner and Cidlowski, 2020; D'Mello et al., 1993).

To identify the upstream triggers of caspase activation, we examined mitochondrial structure and function—key regulators of intrinsic apoptosis (Green and Reed, 1998). HT22 cells treated with saline or PBS displayed significant mitochondrial membrane potential (ΔΨm) depolarization and ultrastructural disruption on TEM. Loss of ΔΨm is a hallmark indicator of mitochondrial permeability transition and precedes the release of pro-apoptotic factors that activate caspase-3 (Kluck et al., 1997; Bernardi et al., 2023). This dysfunction was accompanied by a pronounced decrease in Bcl-2 and an increased Bax/Bcl-2 ratio, a decisive molecular switch that promotes mitochondrial outer membrane permeabilization and apoptosis initiation (Youle and Strasser, 2008; Marzo et al., 1998).

Given the central role of ionic homeostasis in mitochondrial metabolism, we next examined whether oxidative stress and Ca^2+^ dysregulation acted as upstream drivers of mitochondrial injury. Notably, ROS accumulation was markedly elevated in the saline and PBS groups, reflecting a profound compromise in the cellular antioxidant buffering capacity. Elevated ROS is well established to impair mitochondrial function by oxidizing membrane lipids, damaging electron transport chain components, and promoting mitochondrial permeability transition (Zorov et al., 2014). Simultaneously, intracellular Ca^2+^ levels were significantly reduced, consistent with the inability of NaCl-based solutions to maintain physiological Ca^2+^ homeostasis (Morland et al., 2016). In the absence of extracellular Ca^2+^, the reduction in intracellular Ca^2+^ is initiated by the cessation of constitutive Ca^2+^ influx. Whether ongoing Ca^2+^ extrusion via PMCA or NCX contributes to maintaining the lower steady-state level remains to be determined, although both transporters are known to be expressed in neurons and mediate Ca^2+^ efflux under physiological conditions (Brini and Carafoli, 2009; Juhaszova et al., 2000). Although Ca^2+^ overload is classically recognized as a trigger of mitochondrial swelling, ΔΨm collapse, and apoptotic signaling, sub-physiological intracellular Ca^2+^ may also compromise mitochondrial bioenergetics. As a critical regulator of mitochondrial metabolism, diminished Ca^2+^ attenuates the activation of key metabolic enzymes (Rizzuto et al., 2012), thereby destabilizing mitochondrial function and promoting apoptosis (McCormack et al., 1990).

In addition to mitochondrial injury, saline and PBS triggered the excessive activation of stress-responsive signaling pathways. We observed significant phosphorylation of p38 MAPK, its downstream target MK2, and the transcription factor c-JUN, consistent with their established roles in mediating apoptosis under oxidative or metabolic stress (Ki et al., 2013; Wada and Penninger, 2004). By contrast, the absence of NF-κB activation suggests that the cellular stress induced by saline and PBS is pathway-specific rather than globally inflammatory (Liu et al., 2017).

Our finding that calcium-free ACSF markedly reduced HT22 cell viability and induced morphological deterioration underscores the essential role of extracellular calcium in maintaining cellular integrity. This is consistent with the well-established function of calcium in cell adhesion, cytoskeletal organization, and survival signaling pathways (Li et al., 2025). In contrast, supplementation of ACSF with additional pyruvate did not further enhance cell viability or alter cell morphology under our experimental conditions. While pyruvate has been reported to exert neuroprotective effects under certain stress conditions (e.g., oxidative stress or glucose deprivation) (Zilberter et al., 2015), our data suggest that standard ACSF already provides sufficient metabolic support for HT22 cells under baseline culture conditions, and that exogenous pyruvate confers no additional detectable benefit. Collectively, these results indicate that among the components of ACSF, calcium plays a non-redundant role in supporting cell viability, whereas other additives such as pyruvate may be dispensable under normal physiological conditions.

The superiority of ACSF over PBS and saline can be attributed to its more physiological ionic composition and buffering capacity. DMEM and ACSF share a similar ionic profile, including the presence of divalent cations (Ca^2+^, Mg^2+^), glucose, and a bicarbonate-based buffering system (supplemented with CO_2_ in the incubator). In contrast, PBS relies on a phosphate-based buffer and lacks Ca^2+^, Mg^2+^, and glucose, while 0.9% saline has no buffering capacity whatsoever. The observed patterns of cell death, mitochondrial dysfunction, and oxidative stress are therefore consistent with the hypothesis that both adequate buffering and the presence of essential ions (particularly Ca^2+^) are required to maintain neuronal homeostasis.

Several limitations should be acknowledged. First, the lack of direct *in vivo* validation in a surgical model is a major limitation; future studies using rodent models of neurosurgical irrigation (e.g., cortical aspiration or craniotomy) are needed to determine whether the beneficial effects of ACSF observed *in vitro* translate to the complex *in vivo* environment. Second, while we identified calcium as an essential component, systematic dissection of other ACSF constituents (e.g., glucose, magnesium, phosphate) awaits future investigation. Third, the electrophysiological data are descriptive only, due to the inherent variability and depolarized resting membrane potential of the HT22 cell line. These data are presented as descriptive comparisons only and cannot be interpreted as evidence of neuroprotection or directly extrapolated to *in vivo* conditions (Supplementary Figure S2). Despite these limitations, our findings offer a mechanistic rationale for the previously reported *in vivo* benefits of ACSF (Doi et al., 2006; Fujita et al., 2010) by demonstrating preserved mitochondrial function and suppressed oxidative stress.

In summary, our results provide compelling evidence that physiological saline and PBS—owing to their non-physiological composition—fail to support fundamental neuronal requirements and actively promote oxidative, metabolic, and apoptotic stress. In contrast, ACSF offers a physiologically relevant milieu that stabilizes ionic balance, preserves mitochondrial function, and suppresses stress responses. By preserving mitochondrial function and suppressing stress-induced apoptosis, ACSF confers superior neuronal preservation of viability. These molecular insights provide a mechanistic foundation for the superior neuroprotective profile of ACSF observed in previous animal models and clinical investigations (Doi et al., 2006; Mori et al., 2013; Huang et al., 2023), and lay a foundation for future validation studies in rodent surgical models.

## Data Availability

The original contributions presented in the study are included in the article/Supplementary Material, further inquiries can be directed to the corresponding author.
