# Supplementary material for: Artificial cerebrospinal fluid preserves neuronal viability and attenuates apoptosis and oxidative stress in HT22 cells
Source: Front Pharmacol. 2026 Jun 19;17:1833976. doi: 10.3389/fphar.2026.1833976 (PMC13328186; doi:10.3389/fphar.2026.1833976)
Supplement: Supplementary file 1 [file Presentation1.pdf]

# Artificial Cerebrospinal Fluid Preserves Neuronal Viability and Attenuates Apoptosis and Oxidative Stress in HT22 Cells

## Supplementary Material

### Supplementary Methods

### Whole-cell Patch-Clamp Measurements for Electrophysiological Properties

Whole-cell patch-clamp recordings were performed to assess the electrophysiological properties of HT22 cells. Cells grown on 13-mm coverslips were sequentially perfused with serum-free DMEM, ACSF, physiological saline, or PBS in a recording chamber. Recordings were conducted in current-clamp mode using an EPC-10 amplifier controlled by Pulse software (HEKA, Germany). Borosilicate glass microelectrodes were filled with a pipette solution containing (in mM): 117 KCl, 10 NaCl, 2 MgCl□, 10 HEPES, 10 EGTA, and 5 Na□ATP (pH 7.2 with KOH). The extracellular solution was a standard bathing solution (SBS) containing (in mM): 140 NaCl, 5 KCl, 2 CaCl□, 1 MgCl□, 10 HEPES, and 10 glucose (pH 7.4 with NaOH, 290 mOsm adjusted with D-mannitol).

After achieving the whole-cell configuration, current-clamp mode was converted and the holding current was set to 0 pA. Action potentials (APs) were evoked by a depolarizing current injection protocol: a 10-ms, 500-pA pulse preceded and followed by periods of 0-pA holding current (100 ms pre-pulse and 500 ms post-pulse). Key electrophysiological parameters, including the rest membrane potential (RMP), AP amplitude, and AP duration (APD), defined as the time from AP onset to complete repolarization to RMP), were measured from 12 independently recorded cells per condition using offline analysis software (IGOR ProWavemetrics, USA).

**Supplementary table**

**S-Table 1. Ionic composition, osmotic properties, and pH of the four perfusates.**

| Component / Parameter | ACSF | DMEM | PBS | Saline |
|---|---|---|---|---|
| **Ionic Composition (mmol/L)** | | | | |
| $Na^+$ | 145.5 | 154 | 157 | 154 |
| $K^+$ | 2.8 | 5.33 | 4.1 | — |
| $Ca^{2+}$ | 1.15 | 1.80 | — | — |
| $Mg^{2+}$ | 1.1 | 0.81 | — | — |
| $Cl^-$ | 128.5 | $110.0_a$ | 140 | 154 |
| $HCO_3^-$ | 23.1 | 44.05 | — | — |
| Phosphate (total) | $1.1_b$ | $\sim 0.91_c$ | $12.0_d$ | — |
| $SO_4^{2-}$ | — | 0.81 | — | — |
| **Other Key Components** | | | | |
| D-Glucose (mmol/L) | 3.4 | 25 | — | — |

| Component / Parameter | ACSF | DMEM | PBS | Saline |
|---|---|---|---|---|
| Sodium Pyruvate (mmol/L) | — | 0[e] | — | — |
| **Osmolarity (mOsmol/kg)** | 289 | 320–370 | ~300 | ~300 |
| **pH (25°C)** | 7.3–7.4 | 7.0–7.4 | 7.2–7.4 | 6.0–7.0 |

Note: [a] Calculated from NaCl, CaCl□, KCl, and hydrochloride salts of amino acids (e.g., L-arginine-HCl, L-lysine-HCl). [b] Present as KH□PO□. [c] Present as NaH□PO□·H□O. [d] PBS contains 10 mmol/L Na□HPO□ and 2 mmol/L KH□PO□. [e] DMEM (Gibco 11965092) contains no sodium pyruvate. The composition of each solution was based on standard laboratory recipes and commercial product specifications. Osmolarity and pH were measured three times independently; mean values are shown.

## Supplementary Figures

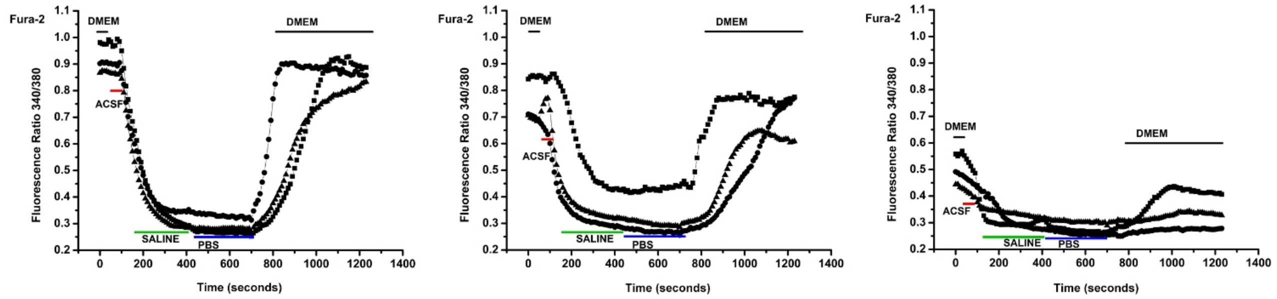


**S-Figure 1. Heterogeneity of intracellular calcium responses to different irrigation solutions at the single-cell level.** Representative single-cell Fura-2 ratiometric traces (340/380 nm) from the dataset summarized in Figure 3B-ii. Cells were sequentially perfused with DMEM (baseline), ACSF, saline or PBS, and then returned to DMEM. Single-cell responses fell into three categories: (Left) robust decrease with substantial recovery (majority of cells), (Middle) moderate decrease with partial recovery, and (Right) minimal change (minority of cells). Most cells showed clear recovery upon re-introduction of $Ca^{2+}$ -containing DMEM, indicating that the heterogeneity reflects differential sensitivity to extracellular $Ca^{2+}$ removal rather than irreversible cell dysfunction. Each trace represents an individual cell. (n = 3 cells per condition).

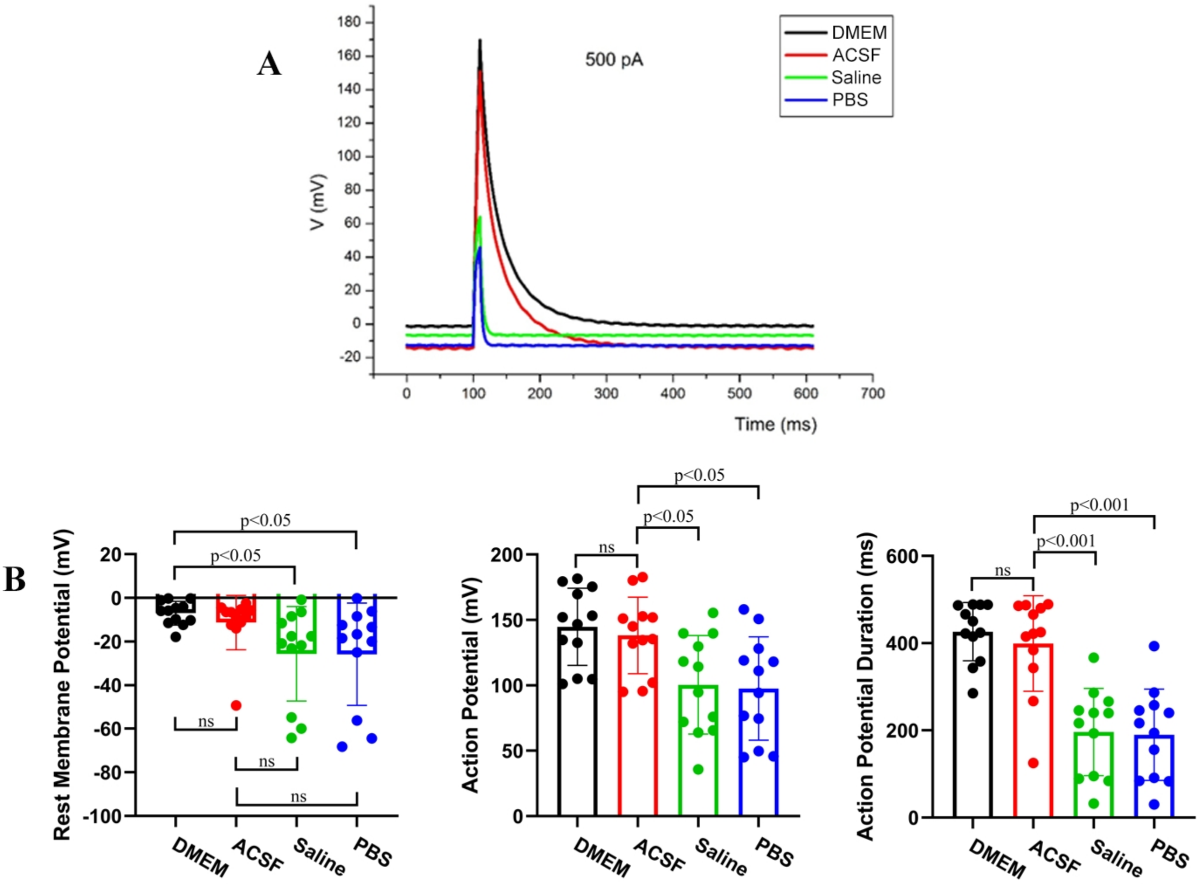


**S-Figure 2. Electrophysiological properties of HT22 cells under different irrigation solutions.** **(A)** Representative action potential (AP) traces in response to a 500 pA depolarizing current step. **(B)** Quantification of resting membrane potential (RMP), action potential (AP) amplitude, and AP duration (n = 12 cells per group, data shown as mean ± SD). RMP values were: DMEM –6.97 ± 5.46, ACSF –11.29 ± 12.46, Saline –25.61 ± 21.70, PBS –25.81 ± 23.51 mV. No statistically significant differences were observed between ACSF and any of the other three solutions ($p > 0.05$ for each pairwise comparison). AP amplitude in the ACSF group (≈138 mV) was comparable to that in the DMEM group (≈145 mV) but significantly greater than in the saline (≈100 mV) and PBS (≈98 mV) groups (both $p < 0.05$). AP duration in the ACSF group (≈399 ms) was comparable to that in the DMEM group (≈426 ms; $p > 0.05$) but significantly longer than in the saline (≈196 ms) and PBS (≈189 ms) groups (both $p < 0.001$).

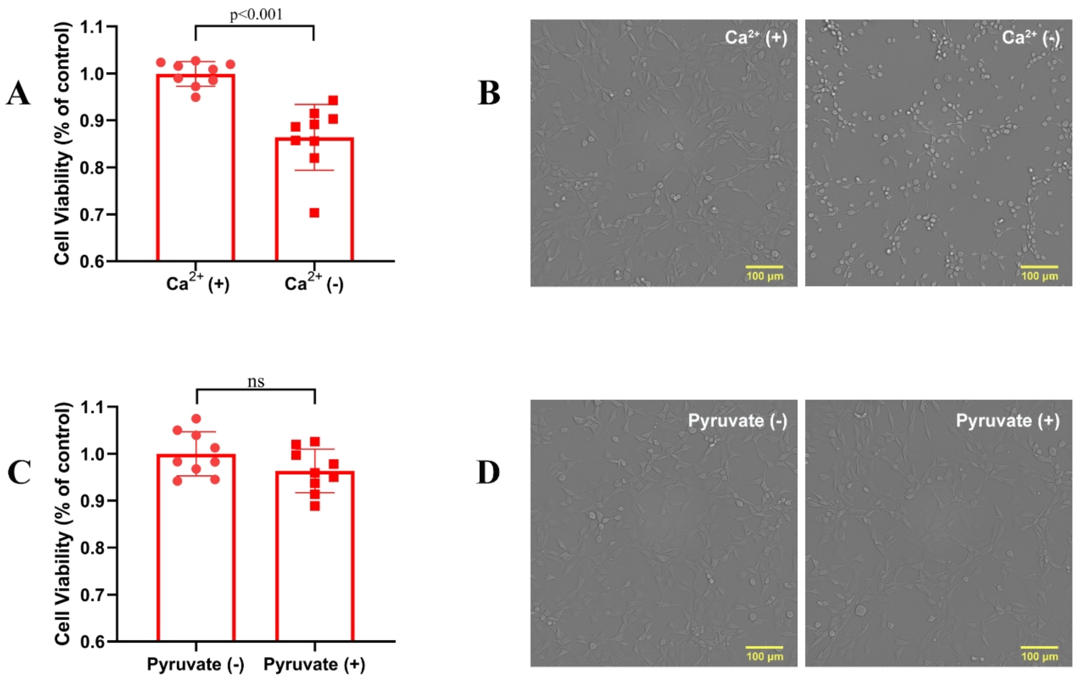


**S-Figure 3. Calcium, but not pyruvate, is required for maintaining HT22 cell viability under ACSF incubation. (A)** CCK-8 assay showing cell viability after 1-hour incubation in standard ACSF or calcium-free ACSF. Calcium-free ACSF was prepared by omitting CaCl□ and adding an equimolar concentration of NaCl to maintain osmolarity. Data are presented as mean ± SD from three independent experiments, each performed in triplicate. $p < 0.001$ vs. ACSF group (unpaired Student's t-test). **(B)** Representative phase-contrast microscopy images of HT22 cells incubated under the same conditions. Cells in standard ACSF exhibited normal morphology with firm attachment and well-spread appearance. In contrast, cells in calcium-free ACSF showed reduced spreading, loss of adhesion, cell rounding, partial detachment, and increased floating cells and debris. Scale bar = 100 μm. **(C)** CCK-8 assay comparing cell viability after 1-hour incubation in standard ACSF versus pyruvate-supplemented ACSF (5 mM). No significant difference was observed ($p > 0.05$, Student's t-test). Data are presented as mean ± SD from three independent experiments (triplicate samples per experiment). ns, not significant. **(D)** Representative phase-contrast microscopy images of HT22 cells incubated in standard ACSF or pyruvate-supplemented ACSF. Both conditions showed comparable cell attachment, spreading, and overall morphology. Scale bar = 100 μm.
